# Resuscitation for Cardiac Arrest and Postcardiac Arrest Care

**DOI:** 10.1155/2020/4053960

**Published:** 2020-05-27

**Authors:** Yan-Ren Lin, Aristomenis K. Exadaktylos, Kee-Chong Ng, John M. Ryan

**Affiliations:** ^1^Department of Emergency and Critical Care Medicine, Changhua Christian Hospital, Changhua, Taiwan; ^2^School of Medicine, Kaohsiung Medical University, Kaohsiung, Taiwan; ^3^School of Medicine, Chung Shan Medical University, Taichung, Taiwan; ^4^Department of Emergency Medicine, Bern University Hospital, Freiburgstrasse, Bern, Switzerland; ^5^Department of Emergency Medicine, KK Women's and Children's Hospital, Singapore; ^6^Emergency Department, St Vincent's University Hospital, Elm Park, Dublin, Ireland

A well-coordinated team, both in prehospital and in-hospital resuscitation, will save lives. For example, high performance cardiopulmonary resuscitation (HPCPR) and extracorporeal cardiopulmonary resuscitation (ECPR), which were both established by closely coordinated teamwork, were recently demonstrated to shorten interruptive time of chest compression and reduce the postcardiac arrest syndrome, respectively [1–4]. Some leading councils (i.e., American Heart Association (AHA) and European Resuscitation Council (ERC)) pointed out the importance of group monitoring in performing resuscitation and emphasized the quality of postcardiac arrest care. Recently, AHA revised the golden guidelines for cardiac arrest resuscitation and postcardiac arrest care on January 2, 2018 [5]. Several leading journals also recently discussed how to increase the quality and outcome in the postcardiac arrest care (including strategic application of ECPR and hypothermia in treating pediatric or traumatic patients), thus making this topic hot and timely [1, 4, 6, 7]. The updated knowledge globally guides the treatment strategies of critical and emergency care. All new guidelines and knowledge can be clinically applied, challenged, and improved upon. In this special issue, we would like to provide an opportunity to introduce various related works discussing resuscitation for cardiac arrest and postcardiac arrest care.

In the field of oxygenation and cardiopulmonary resuscitation (CPR) or respiratory failure, Lee et al. introduced an animal study and demonstrated that continuous flow insufflation of oxygen using a one-way valve resulted in a greater level of oxygenation and less lung and brain injuries than intermittent positive pressure ventilation. Huang et al. performed a meta-analysis study and concluded that administering of high-flow nasal cannula therapy in the emergency department (ED) for respiratory failure patients might decrease the intubation rate compared with conventional oxygen therapy. Cho et al. investigated whether those capnometer readings could be easily affected by fluid exposure during treatment of critically ill patients. They prospectively compared the differences of the ETCO_2_ level between proximal or distal connecting direct connect catheter mount (DCCM) and found that application of DCCM onto the capnometer setting seems to be effective in reducing capnometer malfunctioning under fluid exposing conditions. Lee et al. investigated three-dimensional shapes and deformability of red blood cells (RBCs) during and after asphyxia cardiac arrest in rats. In their study, quantitative phase imaging results revealed that RBC membrane fluctuations, sphericity, and surface area did not change significantly during CPR or after return of spontaneous circulation (ROSC) compared with initial values.

Second, the association between definitive airway establishment and outcomes of patients were also discussed in this special issue. Choi et al. reported that the mean intubation time was significantly shorter in using I-gel blind intubation than using I-gel bronchoscopic intubation or Macintosh laryngoscope.

The other studies focused on the short/long-term outcomes of out-of-hospital cardiac arrest (OHCA) patients. Hsu Chen et al. analyzed the risk of new-onset heart failure of 49,101 nontraumatic OHCA adult patients. They found that, in patients aged from 61 to 75 years, a history of myocardial infarction or cardiomyopathy, and ischemic heart disease or infection as comorbidities, occurring during hospitalization were strong risk factors for new-onset heart failure. However, extracorporeal membrane oxygenation could decrease this risk. Most heart failure events occurred within 60 days after OHCA. For young traumatic OHCA patients, Huang et al. pointed out that Injury Severity Score, Glasgow Coma Scale, and Revised Trauma Score were all associated with increased mortality, prolonged intensive care unit stay, and longer length of hospital stay. Kim et al. administered the Mini-Mental State Examination (MMSE) to 92 cardiac arrest survivors who were treated with targeted temperature management immediately after regaining consciousness. Cognitive impairments were common immediately after patients regained consciousness but recovered substantially before intensive care unit discharge. Moreover, they also reported hypoalbuminemia was common after cardiac arrest, and the serum albumin level at admission was associated with poor neurological outcomes at 6 months after cardiac arrest in patients treated with targeted temperature management. Choi et al. analyzed 6,335 OHCA patients. They found that both the EMS response times to OHCA events in high-rise buildings and the probability of a neurologically favorable discharge differed obviously between homes and public places. The prognosis of an OHCA patient was more likely to be affected by the building structure and use rather than the floor height.

This special issue highlights several articles that report improvements in OHCA-related oxygenation, airway management, resuscitation strategies, and outcomes. We strongly believe that readers will gain useful information from this issue.
